# The Trouble with Triplets in Biodiversity Informatics: A Data-Driven Case against Current Identifier Practices

**DOI:** 10.1371/journal.pone.0114069

**Published:** 2014-12-03

**Authors:** Robert Guralnick, Tom Conlin, John Deck, Brian J. Stucky, Nico Cellinese

**Affiliations:** 1 Department of Ecology and Evolutionary Biology and the CU Museum of Natural History, University of Colorado, Boulder, Colorado, United States of America; 2 CU Museum of Natural History, University of Colorado, Boulder, Colorado, United States of America; 3 Berkeley Natural History Museums, University of California, Berkeley, California, United States of America; 4 Department of Ecology and Evolutionary Biology, University of Colorado, Boulder, Colorado, United States of America; 5 Florida Museum of Natural History, University of Florida, Gainesville, Florida, United States of America; The New York Botanical Garden, United States of America

## Abstract

The biodiversity informatics community has discussed aspirations and approaches for assigning globally unique identifiers (GUIDs) to biocollections for nearly a decade. During that time, and despite misgivings, the *de facto* standard identifier has become the “Darwin Core Triplet”, which is a concatenation of values for institution code, collection code, and catalog number associated with biocollections material. Our aim is not to rehash the challenging discussions regarding which GUID system in theory best supports the biodiversity informatics use case of discovering and linking digital data across the Internet, but how well we can link those data together at this moment, utilizing the current identifier schemes that have already been deployed. We gathered Darwin Core Triplets from a subset of VertNet records, along with vertebrate records from GenBank and the Barcode of Life Data System, in order to determine how Darwin Core Triplets are deployed “in the wild”. We asked if those triplets follow the recommended structure and whether they provide an easy and unambiguous means to track from specimen records to genetic sequence records. We show that Darwin Core Triplets are often riddled with semantic and syntactic errors when deployed and curated in practice, despite specifications about how to construct them. Our results strongly suggest that Darwin Core Triplets that have not been carefully curated are not currently serving a useful role for relinking data. We briefly consider needed next steps to overcome current limitations.

## Introduction

One compelling vision for biodiversity data resources is a “cloud” of interconnected digital objects linked together by an expressive vocabulary describing relationships between the objects, with each object bearing a globally unique identifier that can be resolved using common Internet protocols (e.g., HTTP) (Page, 2009). Thus, humans or computers could learn something about those objects by simply resolving their identifiers via a URI (Uniform Resource Identifiers), which would point to the object itself or a description of the object (e.g., metadata). In this information utopia, data would be unlocked from organizational and technological silos and instead reside in a global data space [1]. One would not “go to the Global Biodiversity Information Facility” or “GenBank” to retrieve species occurrence or genetic data, necessarily, but could traverse from one information artifact related to a physical specimen such as a sequence or image to a whole set of other information about that object contained in linked data stores.

In the biodiversity informatics arena, the vision of a linked and open world of data has foundered on the rocks of a set of deep challenges. Among these challenges are a missing technical infrastructure for easily moving data into proper linked data formats such as RDF (the Resource Description Framework), and a lack of well-established ontologies for properly annotating data in linkable formats [2]–[3]. However, no challenge is more imminently solvable, yet pernicious, than the one surrounding the provision of globally unique identifiers for the biodiversity domain. This view is not simply that of the authors. The Global Biodiversity Information Facility released a 2009 report developed by a global panel of experts whose first words are “GBIF has identified the provision of identifiers for biodiversity objects as one of the central challenges to developing a global bioinformatics infrastructure”, a sentiment shared by others (e.g., [4]).

Although biodiversity informaticians might disagree about which identifier scheme could best solve this “identifiers impediment”, there *is* widespread agreement about the minimum functional requirements for any successful identifiers scheme. These include persistence, resolvability, discoverability, and authority [5]. Persistence is perhaps the most essential requirement, assuring long term availability on the scale of years to decades. Resolvability means that an agent (either a human or computer) can use the identifier to directly discover the data objects or information about those objects [6]. Discoverability refers to the need for identifiers to be clearly described and exposed within and across systems. Finally, an authoritative service assists with identifier curation and standardization, so that like any other digital content, the identifiers remain viable in the long-term. More generally, all four of these critical requirements ultimately relate to how identifiers are curated in actual practice. Even the best identifier schemes will be condemned to failure without sufficient curatorial support. Therefore, we use the concept of “identifier curatorial practice” throughout this paper as the key idea for framing our questions and discussion.

The current landscape related to globally unique identifiers is anything but simple [6]–[7], and the aim of this paper is not to recommend any one solution but to show that the most-used current practice for linking together biodiversity content does not provide a robust solution to current challenges. Before we discuss this issue in more detail, however, it is worth briefly summarizing the recent past and present of the identifiers landscape for biodiversity data. Today, the most widely deployed identifier scheme for biodiversity data is the Darwin Core (DwC) Triplet, which derives from recommendations from the Darwin Core standard ([8]; http://rs.tdwg.org/dwc/terms/). The apparent popularity of the DwC Triplet is certainly not due to a lack of alternatives. The need for an identifier implementation that can meet the requirements discussed above has led to multiple competing possibilities (as discussed in [7]), each with its own proponents. A key, early implementation, the LSID (Life Science Identifiers; [5]), had community support [9], but unfortunately the resources never materialized to build around the LSID standard, and therefore resolver services never caught on. Although DOIs (Digital Object Identifiers), ARKs (Archival Resource Keys), and derivative identifier schemes (e.g., EZIDs) would seem to be a clear alternative given that they are permanent, resolvable, have very strong organizational support internationally, and have long been discussed as potential solutions by our community (e.g., [10]), there have been concerns about scalability and costs, given the potentially billions of data objects that need identifiers. So far, no alternative to the DwC Triplet has reached a level of community consensus, and because the DwC Triplet is derived from fields in the widely-used DwC standard, it is the most common formula for identifiers “left standing” for biodiversity data.

Even though the DwC Triplet has become the identifier scheme most commonly adopted for biocollection specimen records, it was born primarily from a lack of alternatives and was never intended to be a *bona fide* globally unique identifier. It is most often used in the occurenceID term of records in a Darwin Core [8] dataset. The *de facto* recommendation from Darwin Core is the format “urn:catalog:[institutionCode]:[collectionCode]:[catalogNumber]” (http://rs.tdwg.org/dwc/terms/index.htm#occurrenceID). Other repositories that store downstream derivatives of specimen records, such as genetic sequence data deposited to GenBank and the Barcode of Life Data System, also store DwC Triplets as a means to link back to specimens, and they specify similar triplet formats described in more detail later. One possible advantage of the DwC Triplet is that it often directly relates back to the specimen itself as well as any data or metadata records that are related to the specimen. The known drawbacks include problems with global uniqueness, persistence, and resolvability.

Much less clear is how well DwC Triplets are deployed in the wild. Do current curatorial practices lead to the creation of DwC Triplets that serve the needs of linked data? This question is of immediate importance because the DwC Triplet is often the only identifier associated with specimen records, and because it is also accepted as a standard by other platforms that create and store downstream derivative sequence data, such as the Barcode of Life Data System ([11]; http://www.boldsystems.org/). The goal of our study was to determine how the DwC Triplet is used in practice and whether currently available DwC Triplets are sufficient to link digital biodiversity records from one data publishing platform (e.g., VertNet; [12]) to records from another platform (e.g., Barcode of Life or GenBank).

The key questions we tackle are:

1. Are Darwin Core Triplets consistently formatted when published as part of specimen record datasets in VertNet and are they consistently formatted in downstream aggregators such as the Barcode of Life Data System and GenBank? How often do we find exact matches across repositories for Darwin Core Triplets?
2. How often do records have a DwC Triplet that includes all required information, but is not constructed according to the recommendations in the standard guidelines? For example, incorrectly formed DwC Triplets might use different delimiters or switch the order of the individual components.
3. How often do records contain a doublet as opposed to a triplet; e.g., an identifier that is missing the “collection code” part, and is thus expressed as “ [institutionCode]:[catalogNumber]”? How often do doublets point back to a specific VertNet record or instead point back to multiple possible records, in cases where an institution has multiple resources published?

By quantifying these issues for the first time, we hope to raise awareness about where the community stands regarding not only theories about which identifiers make the most sense for the future, but the immediate curatorial realities about where we are now.

## Materials and Methods

We gathered and analyzed Darwin Core Triplets, or clear variants of DwC Triplets, from vertebrate specimen and sequence data records from three repositories: VertNet (vertnet.org), GenBank (http://www.ncbi.nlm.nih.gov) and the Barcode of Life Data System (BOLD; http://www.boldsystems.org/), which is a workbench that also submits data records to GenBank. Syntactically correct DwC Triplets were recognized using regular expression text matching. A more complex text parsing algorithm was used to recognize variants of DwC triplets that did not follow the recommended syntax. Next, all DwC Triplet-based identifiers were categorized according to how complete they were (e.g., a triplet or doublet) and how they were formed syntactically. Finally, we attempted to match identifiers across repositories and analyzed the precision of cross-dataset identifier matches. Each of these steps is described in detail below.

### Recognizing Darwin Core Triples in Datasets

Prior to dataset assembly, we specified rules for documenting DwC Triplets in fields where they are likely to be stored given existing best practices. Below we discuss these rules in more detail and then describe each dataset we assembled. Well-formed DwC Triplets contain Institution Code (IC), Collection Code (CC) and Catalog Number (CN) for each record and use a colon “:” to separate them. We consider these to be canonical DwC Triplets, which have the best hope of fulfilling their intended purpose. Non-canonical or “coerced” variants of these 'structured vouchers' range downward in interpretability and usefulness and fall into several categories. The first type has all three pieces of information (IC, CC, CN,) but does not follow canonical structure. The next category only lacked its collection code, forming a “doublet”. A final catch-all category includes those identifiers with multiple issues, which often consisted of nothing more than a plain Catalog Number.

Text was recognized as a canonical DwC Triplet if it followed these three rules:

1. <2–8 capital letters for an institution code>:
2. <Word with leading capital for collection code>:
3. <any numeric or string containing a number for a catalog number>

A regular expression to accept canonical DwC Triplets is “[A-Z]{2,8}\:[A-Z][a-z]+\:.*[0-9]+.*”.

We also identified DwC Triplets that did not follow the canonical structure using the following algorithm, given the rules above:

1. Break a text value suspected of containing a DwC Triplet into fragments by semicolons and open parenthesis (which were most commonly used to separate multiple identifiers within a single field).
2. Partition each fragment into tokens by [“:”, “.”, “-“, “_”, “#”, “ ”] (colon, dot, hyphen, underscore, hash or space) and then:
  - If the final token is an all-caps word or a multi-digit-number, use it as a default IC or CN respectively, if an IC or CN is not found in the following steps.
  - If the first three tokens match the rules for IC, CC and CN, use them as such.
  - Else if the first two tokens match the rules for IC and CN, use them as such and do not assign a CC.
  - Else if the first two tokens match the rules for IC and CC, use them as such along with the default CN, if available.
  - Else if the first two tokens match the rules for CC and CN, use them as such along with the default IC, if available.
  - Else if the first token matches the rule for CN, use it as such along with the default IC, if available.
3. If a record with multiple identifiers has any DwC Triplet with an institution code, use that IC as the default for any other identifier lacking an IC.

### VertNet data assembly

URLs for DarwinCore Archives - a data delivery format described in more detail in [13] - were taken from http://ipt.vertnet.org:8080/ipt/(mid-August 2013) and the resulting files were loaded into a PostgreSQL (http://www.postgresql.org/) database. The VertNet data files were harvested from the core VertNet IPT instance and not from independent VertNet providers. We assembled Darwin Core Triplets according to best practices, focusing on the relevant Darwin Core record-level terms “institutionCode” and “collectionCode,” and the occurrence-level term “catalogNumber”. These fields were later joined to reconstitute a well-formed triple for each record.

### GenBank data assembly

We initially attempted to collate all GenBank records from a subset of 24 institutions in common with VertNet. We later refined our approach to develop more permissive filters over a subset of GenBank records which excluded GenBank divisions with many records never submitted as vertebrate vouchers (e.g., bacteria divisions). In short, we fetched all of GenBank's primate, rodent, mammal, and “other vertebrate” divisions (PRI, ROD, MAM, VRT) totaling 2.94M records (records were downloaded from: ftp://bio-mirror.net/biomirror/genbank/early Nov. 2013). We then filtered these records by first checking for existence of the specimen_voucher field, parsing and then removing those records that were clearly not from vertebrate biocollections based on expert-opinion of two of the authors (Cellinese and Guralnick) vetting a list of institution codes from the Global Registry of Biorepositories (http://grbio.org). The International Nucleotide Sequence Database Collaboration (INSDC) specifies a format for this field (http://www.insdc.org/controlled-vocabulary-specimenvoucher-qualifier) in the form “[<institution-code>:[<collection-code>:]]<specimen_id>” which is syntactically similar to the Triplets in other aggregators such as VertNet and BOLD. The specimen_voucher field was parsed to generate presumptive IC, CC, and CN fields, which were assembled into triplets following the canonical pattern and loaded into a PostgreSQL database. The parsed IC, CC, and CN fields were finally concatenated to reconstitute a well formed triple for each record.

Approximately 80% of the vertebrate records we collated lacked a “specimen_voucher” field in the feature table, leaving ∼595,000 records with some form of candidate for a voucher identity. Before accepting these ∼595,000 records as truly representative, we examined other fields that could contain a voucher identity that did not overlap with the set of records with a specimen_voucher field. In addition to looking for DwC Triplets we also checked for other forms of identifiers including universally unique identifiers (UUIDs), life science identifiers (LSIDs), digital object identifiers (DOIs) and other variants such as EZIDs from the California Digital Library (http://ezid.cdlib.org/) but located none from our pool of records.

We considered several other GenBank data fields as possible sources of DwC Triplets, but ultimately did not include any others in our analysis. Many records with a specimen_voucher field also had the same DwC Triplet expressed in the DEFINITION field. However, the number of distinct candidate records added by examining extra fields would have been trivial - under 1000. The ‘isolate’ field in the feature table was also considered, as there were ∼90,000 records in common with those with a specimen_voucher field. Although the vast majority of isolate values were distinct, further inspection showed these to mainly be names of genes, and few could be interpreted to be anything resembling a DwC Triplet (total number, 487). The feature table fields ‘db_xref’ and ‘note’ were also considered and not found to be viable sources of DwC Triplets.

### GenBank data set assembly – Linkout data

The “Linkouts” (http://www.ncbi.nlm.nih.gov/projects/linkout/) visually presented when browsing a page at GenBank are not included in their readily available bulk download files, necessitating additional effort to collect them as they are an apparent source of DwC Triplets. We also thought that we might be able to use the Linkout URL as a hint to resolve which CC or IC to assign in certain cases. Data on linkouts were assembled by querying NCBI's Web API Eutils with our list of 595,000 mined GenBank accession records having some value in the specimen_voucher qualifier field (see above). For more details on using NCBI's Eutils see http://www.ncbi.nlm.nih.gov/books/NBK25499/. We first queried the Epost service with our GenBank Accession (LOCUS) ID; subsequently, Elinks calls were made to map our accessions to GenBank's GenInfo ID (GI) sequence number, and then the GI number was used to associate Linkout information with our accessions. The results were returned via an ESummary call. To get the presumptive specimen identifier from a Linkout, we split the end from the URL instead of looking in the linkname field,

since the URL field always exists, and the more specific alternative depends upon the whim of the submitter populating the field. The split was made by isolating whatever is after the final slash “/” and then whatever follows the “ = ” sign (if one exists). This strategy worked well for this set, but it is a brittle approach as URLs need not be in their canonical form to function as browsing links.

### BOLD data set assembly

BOLD records for Chordata were downloaded from: http://www.boldsystems.org/index.php/Public_SearchTerms?query=Chordata[tax] June 2013. BOLD specifies that specimen data submission must precede submission of genetic sequence data and is clear about the protocols and fields specifications for those submissions (http://www.boldsystems.org/index.php/resources/handbook?chapter=3_submissions.html&section=data_submissions). MuseumID (called catalognum in their download file) is the field allocated for the specimen identifier, and is specified to contain a Darwin Core Triplets of the format “Institution acronym:collection code:catalog number”. However, SampleID may also contain a DwC Triplet and therefore we checked both fields. We found simpler cases where only one DwC Triplet existed or the contents of the fields agreed, and the more interesting cases where they did not (just under half with both columns populated were not exact matches). DwCTs were constructed following the routines described above, in the “GenBank data assembly” section.

### Determining matches across repositories

We determined exact matches where all three components were present and identical, along with less precise matches (triplets to doublets and doublet-doublet matches). We used shell scripts in the filesystem and SQL in a PostgreSQL relational database to ascertain matching DwC Triplet between repositories. Matching in the filesystem used common UNIX commands for creating and manipulating files. Matches generated from the filesystem were verified using SQL joins on the PostgreSQL database.

### Data and code availability

All data and code used in this project are available on GitHub (https://github.com/TomConlin/trouble_w_triples). To more easily reproduce our analyses, we have made the data, source, and functional environment available as a Docker (http://www.docker.com/) container (available here: https://registry.hub.docker.com/u/tomc/trouble_w_triples/). All the data is also available in Dryad (http://dryad.org) Data identifier: doi:10.5061/dryad.4b115 for archival purposes. Full documentation can be found at the sites provided above. New experiments and improvements are encouraged.

## Results

The main results are summarized in three Tables below. Table 1 shows the total number of records examined for each repository, the number of Darwin Core Triplets that were discovered within the repositories, and a further breakdown into two categories: canonical and coerced. Canonical triplets are those that are considered perfectly well-formed according to best practices and coerced are those that had either missing data or improper syntax.

**Table 1:** Summary of DwC Triplets per repository.

Repository	Total records examined	Records w/specimen info.	Canonical DwC Triplet	Coerced DwC Triplet
VertNet	8,216,424	8,216,424	8,214,727	1,697
GenBank	2,940,092	595,646	10,487	281,966
BOLD	216,809	216,809	1,330	63,350

A canonical DwC Triplet is a triplet that is complete and conforms to standard representation. A “Coerced” DwC Triplet is one that is either missing a part of the triplet (e.g., a collection code) or in a non-standard syntax.

We then further break down coerced Darwin Core Triplets into three classes shown in Table 2. The first class represents identifiers that only have syntactic problems (e.g., the format is not according to the recommendation in the standard). The second class represents those that only have missing information (e.g., lack of collection code), and the third are those with both kinds of problems.

**Table 2:** Irregularities in expression of Darwin Core Triplets broken down by class of issue.

Repository	Syntactic only irregularities	Missing data only irregularities	Both syntactic and semantic problems
VertNet	0	1,697	0
GenBank	1,984	93,947	186,128
BOLD	986	4,564	58,843

**Figure 1:**
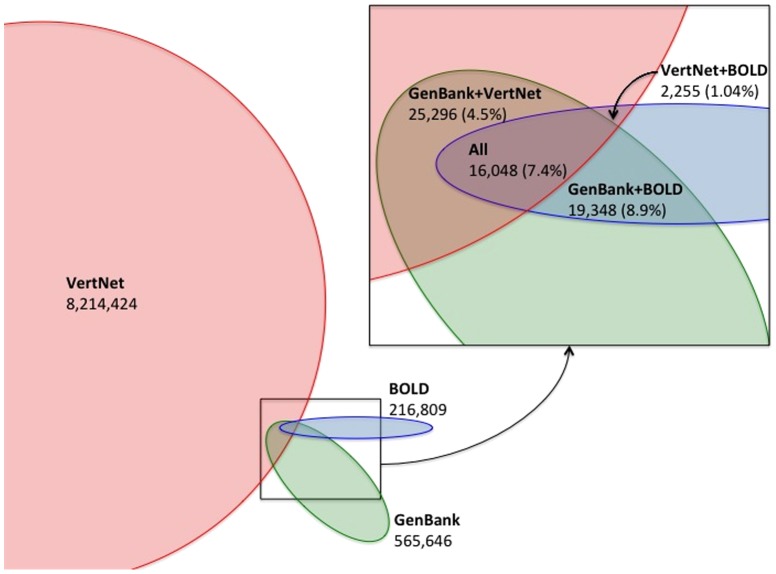
A schematic representation showing proportional numbers of Darwin Core Triplets – represented as different sized ellipses - across repositories and the overlap between them. The inset shows the overlap regions in more detail. The numbers associated with each repository and areas of overlap are for all types of matches, not just triplet-to-triplet matches. The percentages represent the number of matches between two (or three repositories) divided by shared triples in the smallest of the two (or three) polygons.

Finally, we compiled information about matches between repositories. In Table 3, the “canonical” matches are exact matches where both repositories have canonical DwC Triplets. Triplet-Triplet reflects matches where the triplets were not syntactically standard. Triplet-Doublet matches are cases where there is a triplet in one case and a doublet in the other (both canonical and syntactically nonstandard triplets were used), and doublet-doublet matches are where collection codes are missing for both DwC Triplets. A common situation in linking occurrences between data sets is finding a single occurrence record in VertNet and multiple sequences describing different loci in GenBank. The parenthetical numbers represent distinct DwC Triplets shared between repositories. There were 16,048 effective DwC Triplet matches across all three repositories. These results are also summarized in Figure 1, which graphically represents the proportion of records and matches.

**Table 3:** A breakdown of matches and match types across repositories.

Match type	Canonical	Triplet-Triplet	Triplet-Doublet	Doublet- Doublet
VN-BOLD	0	103	30,161 (18,766)	0
VN-GenBank	2,179	2,392	104,027 (58,116)	0
BOLD-GenBank	462	67 (60)	69 (30)	283,875 (27,916)

Parentheticals give the number of unique DwC Triplets involved in the matches.

Linkout data represent one final way that GenBank records can be linked back to specimen records. After loading the Linkout data into the database, we were able to analyze the GenBank Linkout data with a specimen_voucher association. There were ∼86,000 accessions with associated Linkout information, and of these, the vast majority pointed to the Dryad Digital Repository (http://datadryad.org) (∼73,000). Dryad hosts data sets from which sequences were derived and therefore, the Linkouts technically do not reference connections to sample-based instances. Additionally, out of the accessions with Linkouts from Dryad, there are only 93 distinct URLs. Arctos (http://arctos.database.museumhttp://arctos.org/; a web-based database system; 7,193 Linkouts) and BOLD (6,236 Linkouts) accounted for most of the remaining Linkouts, with the Smithsonian Fish Collection having 126 and University of Kansas Natural History Museum having 1. Thus, we found that the use of Linkouts in association with specimen_voucher information is limited to a few systems promoting their use.

## Discussion

The great value of the Darwin Core Triplet is its apparent simplicity and direct link to the specimen itself. Most objects in natural history museum collections bear a catalog number, either directly on the object or located in proximity if those specimens are lot-based. If specimens are subsampled for tissues that generate genomic DNA or targeted sequences stored in repositories such as the Barcode of Life Data System or GenBank, these same identifiers propagating with the subsampled material or output sequence files promise the ability to link back to these vouchers.

We examined data records for an iconic and well-studied clade of organisms, the Vertebrata, to determine how often and how well Darwin Core Triplets are used to help make those linkages across repositories. Our results suggest that DwC Triplets, when present, have hugely varying levels of quality, based directly on the source dataset. Table 1 shows a large percentage of canonical triplets for VertNet and much lower success for both GenBank and BOLD. The difference here lies in the fact that we harvested VertNet records from an Integrated Publishing Toolkit (discussed in [13]) instance representing data that were curated under the supervision of data managers. GenBank and BOLD represent data that were input independently by many different researchers and without mechanisms to assure compliance or curatorial oversight. We should note that in looking at those VertNet data that are not part of the curated IPT instance from which we harvested, the Darwin Core Triplets were not nearly as consistent or well-formed as their curated cousins.

The problems with the Darwin Core Triplet have long been noted from first principles (http://iphylo.blogspot.com/2011/12/dna-barcoding-darwin-core-triplet-and.html), but what has not been nearly as clear is just how idiosyncratically Darwin Core Triplets are deployed in practice. Although documented specifications have been developed, researchers are still often confronted with only the barest of guides and few tools to populate specimen or voucher_id fields in repositories such as BOLD and GenBank. Many are clearly doing the best they can, and it is worth noting that many are taking seriously the process of entering information into voucher_id fields. However, in lieu of validation processes, the question is: how well is this working? Our results show that there is a shockingly small set of Darwin Core Triplets that are well-formed in GenBank and BOLD.

We also note a dismally small number of Darwin Core Triplet matches across repositories, especially given that BOLD best practices require specimen vouchers and that these propagate to GenBank. What we find instead is that the vast majority of matches (>99%) are triplet-doublet or doublet-doublet matches. The problem is that such matches are often ambiguous. If an institution has more than one collection published, the doublet is no longer necessarily even locally unique and can point, therefore, to records in multiple collections.

To further exacerbate these problems, both GenBank and BOLD offer multiple solutions for providing links to specimen records and provide little detail about which method is preferred. In BOLD, both catalognum and sampleid fields can contain Darwin Core Triplets. Deciding if one, both, or neither contain usable identifiers for linking to specimens or other digital or information derivatives is difficult to implement programmatically. Out of the ∼4000 BOLD records that had different contents in the two possible fields, careful parsing of both fields only increased the number that certainly matched each other by ∼100. GenBank provides multiple fields where identifiers may be located along with a wholly separate mechanism for links using its “Linkout” mechanism. Although the direct URL link to a specimen record is valuable for immediate access, it is also fraught with the problems of impermanence. As in BOLD, it also can lead to inconsistencies that can be hard to reconcile.

Linkage problems immediately impact the ability to ask very simple questions that should be trivial to answer, e.g., “Which specimens in VertNet have associated sequences in GenBank?” Instead, the question becomes, “Can we assemble Darwin Core Triplets and attempt comparisons to potentially locate close matches?” In some cases, there is not enough information to make any assessment at all, and in others it is achievable to narrow the field to a small number of possible specimens from the same institution bearing similar catalog numbers but unclear or missing collection codes.

We assembled this snapshot of current practices during a period when publishing best practices are starting to shift, at least for Darwin Core formatted records. Organizations such as iDigBio and GBIF are coming to terms with allowing providers to mint their own identifiers. GBIF has enabled providers to use the occurrenceID field as the core identifier in Darwin Core Archives and iDiGBio has published a set of “Guidelines for Managing Unique Resource Identifiers” (https://www.idigbio.org/sites/default/files/videos/slides/iDigBio_URI_recommendation.pdf). While we strongly support these changes, we just as strongly advocate for best practices and an aligned approach to identifiers being properly used in the occurrenceID. We have found that in the absence of curatorial supervision from a data manager, Darwin Core Triplets are largely useless in linking data and thus changes implemented by the large data aggregators to accommodate identifiers must be accompanied by services for validating, minting, and resolving them, whether the identifier is a DOI, ARK, or a Darwin Core Triplet.

Given the results from this work, and clear messages coming from data publishing specialists (http://gbif.blogspot.dk/2014/04/ipt-v21.html), we argue strongly for a two-pronged approach. First, we advocate for better curatorial practices related to the Darwin Core Triplet. These can include stronger focus on compliance and validation when Triplets are entered into data systems, and better minting and resolving systems that can take the burden off the data producers. Second, we see value in testing deployment of other identifier schemes, which may have less curatorial overhead. The Darwin Core Triplet requires effort by producers to correctly assemble three pieces of metadata; other schemes where minting and maintaining persistence is simpler may be more efficient in the longer term. Even so, *any* identifier scheme still must be carefully minted and validated, with recognition that, much like social security numbers, DUNS codes, product barcodes, and vehicle identification numbers, they are valuable assets in tracking and managing specimens in a global biodiversity linked data web. Only then can we gain more traction to rediscover the linkages between growing repositories of specimens, tissues, sequences, and other digital data derivatives.
